# Purchases of Meats and Fish in Great Britain During the COVID-19 Lockdown Period

**DOI:** 10.3389/fnut.2021.648160

**Published:** 2021-06-01

**Authors:** Cesar Revoredo-Giha, Carlo Russo

**Affiliations:** ^1^Rural Economy, Environment and Society Department, Scotland's Rural College, Edinburgh, United Kingdom; ^2^Department of Economics and Law, University of Cassino, Lazio, Italy

**Keywords:** UK meat market, consumers' response, COVID-19 pandemic, nutrition quality, UK diet

## Abstract

The purpose of this paper is to provide an analysis of the purchases of meat and fish in Great Britain during the lockdown period using time series constructed from a unique scanner panel dataset available since 2013 and which is based on information about 30 thousand households. The time series available for the analysis represent the purchases (expenditure and quantities) of all consumers and by income groups were used to compute price and quantity indices all the meats together and for each meat (i.e., beef, lamb, pork, poultry, and other meats) and fish. The changes in expenditure were decomposed into changes in prices, quantities purchased and changes in quality purchased (trading up/down in quality) i.e., whether cheaper meat or fish were purchased. A further extension of the analysis was produced by considering the evolution of calories, saturated fats and sodium per purchased quantity for meat and fish during the period of study. The results indicate that although the shares of quantities remained relatively constant, the calories, saturated fats and sodium from the purchased quantities showed an increasing trend, indicating that most of the incomes groups were lowering the nutritional quality of their meat and fish purchases. This is clearly shown by the fact “other meats” represents on average 39 percent of the calories contributed by meat and fish, 49 per cent of the saturated fats and about 68 of the total sodium in meat and fish during the lockdown period. This result highlights the need to emphasize healthy messages related to the purchases of meat.

## Introduction

There are several reasons why the analysis of the purchases of meat is important. Some of them are from a nutritional point of view and another is from an environmental one. From the nutritional side, according to the NHS (1) meat is a good source of protein, vitamins and minerals in the diet and eating meat can be part of a healthy and balanced diet (2). In fact, a balanced diet can include protein from meat, as well as from non-animal sources such as beans and pulses.

It is important to note that some meats are high in saturated fat, which can raise blood cholesterol levels. High consumption of red and processed meat has been linked to bowel cancer (1). Processed meat refers to meat that has been preserved by smoking, curing, salting or adding preservatives. This includes sausages, bacon, ham, salami and pâtés. As a reference, 90 g is equivalent to around three thinly cut slices of beef, lamb or pork, where each slice is about the size of half a piece of sliced bread. A cooked breakfast containing two typical British sausages and two rashers of bacon is equivalent to 130 g (1). Thus, the NHS advice is to reduce the cooked weight of red and processed meat a day down to 70 g, which is the average daily consumption in the UK (1).

Meat consumption has gathered plenty of attention due to the environmental impact of animal production (3–5). There is an increasing interest in more environmentally friendly food production as shown by the report from the Assembly Citizens (6) chapter 6 about the food we eat and how we use the land. The report indicated that members of the assembly were willing to lower meat and dairy consumption by 20–40 percent by 2050.

The COVID-19 period, i.e., since March 2020, is interesting one to analyse consumption because (at least at the beginning) households shifted toward supermarket purchases due to the food service closure and also because literature has indicated changes in consumption habits [e.g., (7, 8)]. Using the latest figures from UK Department of Food, Environment and Rural Affairs (Defra) Family Food annual report (9), if the lockdown would have been perfect, and all the meals had been taken at home (i.e., all the money is still spent on food), it would have implied a maximum average increase in demand for household supplies of around 44 percent; this is, of course, an average figure with the first income decile (i.e., the least affluent group) being able to spend 24 percent more on their household food items and the last decile (i.e., the most affluent group) 66 percent (10).

The purpose of this paper is to provide an analysis of the meat purchases during the COVID-19 period (i.e., March to June 2020) using time series derived from a large panel dataset, the Kantar Worldpanel dataset. In particular, the study is interested to study the evolution of different meats and their nutritional contribution considering the purchases of households of different income groups.

The underlying dataset, from where the available time series used in this analysis come from, comprises grocery purchases for about 30,000 households in Great Britain (the dataset does not contain information for Northern Ireland). The time series, which were available since 2013, were constructed using about gross up weights at the level of purchases and also households classification by income groups. It is important to highlight that the Kantar's information (as well as Defra's) refer to food purchases and not to actual consumption although it is common to call them by both names given the close association between them.

The structure of the paper is as follows: it starts with a review of the purchases of meat and fish in the UK based on the publicly available dataset [e.g., Defra's Family Food, (9)], which provides annual information by food categories from 1974 until 2018; next, the methodology and data are presented, followed by the results and discussion.

## Purchases of Meat and Fish in the UK

According to United Nations Food and Agriculture Organisation (FAO) data (11), the biggest meat consumers in the world are those in the U.S. where 98.6 kg was consumed per person in 2017. Britain's meat consumption at 61.4 kg per person per year is similar to other European countries such as Ireland, France and Germany.

Due to its importance for the food sector, several papers have addressed the demand for meats in the UK focusing on the importance of price, income and household variables (12–14). Additionally, other papers have modeled meat and fish demand as part of the demand for food [e.g., (15)]. However, the purpose of these studies has been to study the response of meats to changes in prices and income and not the evolution of the purchases of different meats and their contribution to nutrients due to a massive exogenous shock such as COVID-19.

**Figure 1:**
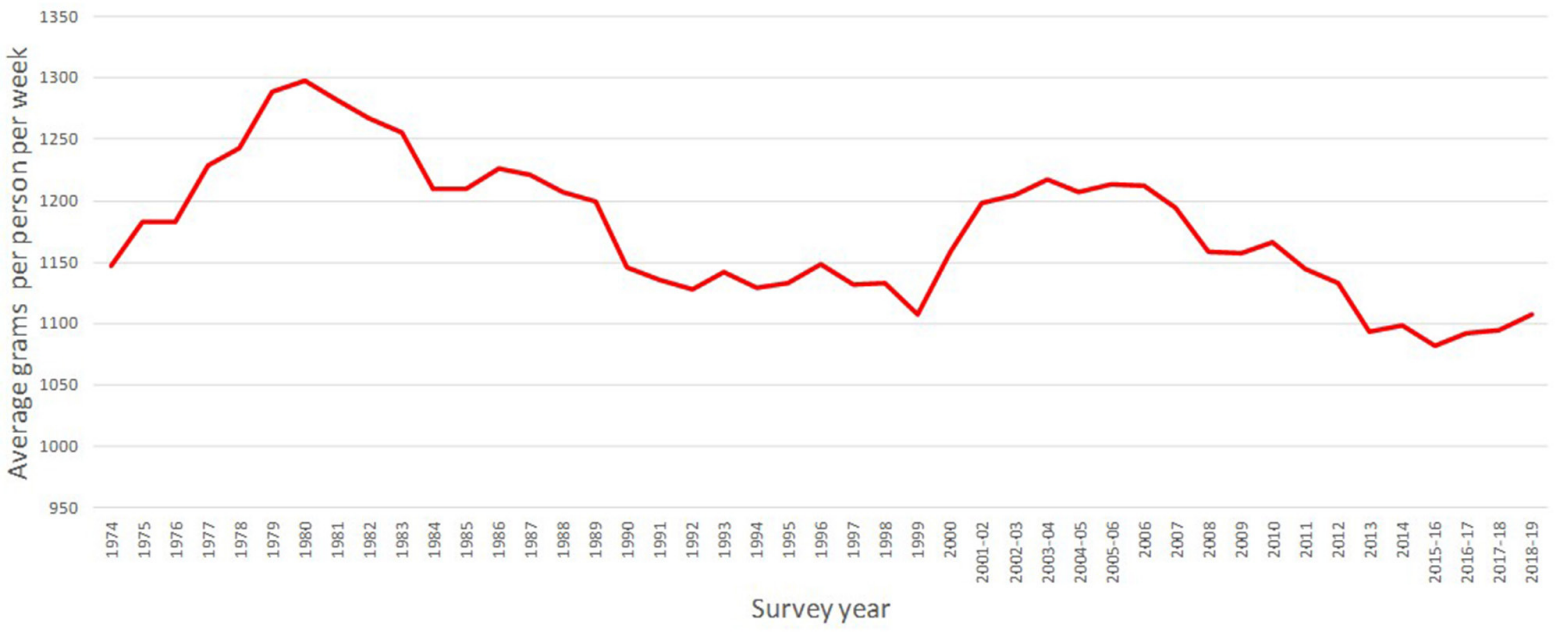
United Kingdom—Per capita meat and fish quantities purchased, 1974 to 2018-19. Source: Defra's family food.

Defra's Family Food (Defra's, 2020) provides time series information about the average per capita quantities purchased per week from 1974 to 2018-19. This information is collected from the Living Costs and Food Survey (former Expenditure and Food Survey). This is a survey conducted by the Office for National Statistics (ONS) and the Department for Environment, Food and Rural Affairs (DEFRA) which collects data about private household expenditure and quantity purchased in the United Kingdom from a sample of about 5–6 thousand households. Figure 1 presents the purchases of all meat and fish together over time.

**Figure 2:**
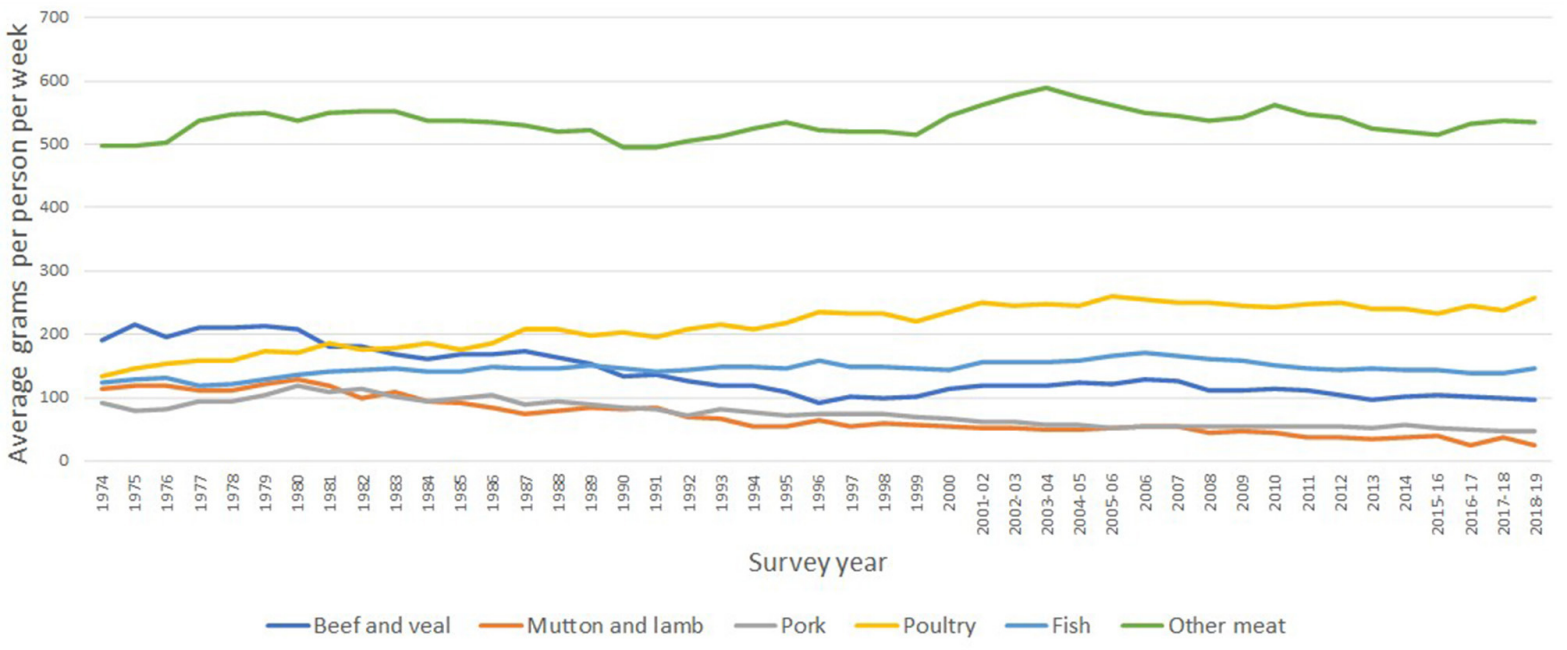
Per capita quantities purchased of meat and fish, 1974 to 2018-19. Source: Defra's family food. Note: Other meat also includes takeways and ready meals that contain meat.

Figure 1 shows there are no single trends on the series, but it can be broken by periods. Thus, there is an increase in the purchases of meat from 1974 to 1980, followed by a decreasing trend from 1980 until 1999, where the purchases decreased from about 1,300–1,100 g. After a short period of non-linear increase up to 2005-06, the series decreased to 1,082 g to finally present an increasing trend. Since as shown in the introduction not all the meat has the same nutritional contribution, it is important to break them down into the different types of meats. These are presented in Figure 2.

The first fact shown in the figure is the importance of other meats on the total purchases. This group includes burgers, sausages, offals, meat takeaways. This processed meat group is an important contributor of calories and saturated fats (unfortunately Defra's data only presents information about the nutritional content of the entire purchases and not the contribution of each component).

The second fact in Figure 2 is the increasing trend on the consumption of poultry (particularly chicken) from 133 g in 1974 to 258 according to the 2018-19 survey. By contrast sheep-meat (mostly lamb) consumption has seen a gradual decline. According to Savills Co (16) this is likely due to limited cooking versatility and a perceived higher price point. The trends on beef and pork consumption, as shown in Figure 2, are similar to that observed for lamb. It is possible that these negative trends on red meat are associated to changes in consumption patterns due to healthy messages.

The third fact is that the per capita consumption of fish, as shown in Figure 2, has remained relatively constant over time, representing an average of 146 g per week.

Note that the alternative protein sector (plant-based meat substitutes) is not considered here due to the fact that despite the increase in its market it still represents <1 percent of the meat market (16).

The other source of information on the UK consumption is the National Diet and Nutrition Survey (NDNS). The NDNS rolling programme is a continuous, cross-sectional survey. It is designed to collect detailed, quantitative information on the food consumption, nutrient intake and nutritional status of the general population aged 1.5 years and over living in private households in the UK (17). The survey covers a representative sample of around 1,000 people per year. Fieldwork began in 2008. It is important to note that NDNS statistics refer to food consumption in contrast with Defra's Family Food, which refers to food purchases.

According to the NDNS, the intake of red and processed meat is continuing to fall. For teenagers, there was a decrease of 15 g a day over the 9-year period. However, the average consumption for adult men remains above the recommended maximum of 70 g a day.

## Materials and Methods

### Data

The data used in the analysis were time series from the Kantar Worldpanel dataset for Great Britain for the period 2013 to 2020. The underlying dataset, i.e., from which the time series were constructed collects information about purchases at the level of actual products by about 30 thousand households. The data exclude food for out-of-household consumption. The time series were constructed by Kantar using gross up weights that allow to compute country-level- time series.

It should be noted that Kantar data use months of 4 weeks (i.e., a year is made of 13 months), therefore, the dataset starts in 2013 (week commencing the 6th of January) and end in 2020 (week commencing the 12th of July) comprised 98 observations. For the analysis defined five periods of interest were defined. The pandemic outbreak was from February 24 to March 22, 2020 (the first death from Coronavirus in the UK was confirmed on March 5). For easy of reference, this period is defined as period t. The first lockdown period (or period t+1) was from March 23 to April 19, as the UK government enforced restrictive measures starting March 20. The second lockdown period (or period t+2) was from April 20 to May 17 (as the UK government started easing restrictive measures at the very end of this period). The first and the second post-lockdown periods went were from May 18 to June 14 (period t+3) and from June 15 to July 12 (period t+4), respectively.

The household data contain information about their income ranges (i.e., £0–29,999, £30,000–39,999, £40,000–49,999, £50,000–59,999, £60,000 - over) and it was used to estimate purchases time series by income group in per capita terms. The income ranges were provided by Kantar.

The meat products were aggregated based on Kantar World panel categories. Thus, beef refers to fresh and frozen beef cuts (it also includes chilled burgers, unlike Defra's survey in Figure 2; frozen burgers, which is a marginal category was classified as part of other meats); lamb (fresh and frozen lamb meat); pork (fresh and frozen pig meat); poultry (fresh and frozen poultry meat); fish (fresh and frozen fish) and other meats (made of the remaining meats, which are mostly sausages, bacon, and offal). In contrast with Defra's data it does not include takeaways because Kantar does not collect that information. The dataset also includes nutritional information at the level of product (in contrast to food category as in the case of Defra's Family Food). This is collected from the back or side of packaging nutrition information. Therefore, the available nutrients in the dataset were calories, proteins, carbohydrates, sugar, fats, saturates, fiber and sodium. In this study, the focus was on calories, saturated fats and sodium.

### Methods

The methods start with the evolution of the expenditure, prices and quantities purchased of meats and fish. It is followed by the decomposition of expenditure during the COVID-19 period, and finally, a trend analysis is carried out to test whether the purchase of calories, saturated fats and sodium during the COVID-19 emergency period was different to the ones from previous periods.

#### Price and Quantity Index Numbers

The methodology consisted of constructing time series for the period 2013 to 2020 for meats (beef, lamb, pork, and poultry) by income group. As expenditure shares change over time Tornqvist-Theil-Divisia (TTD, hereafter) price and quantity indices (18) were produced. These indices are a weighted geometric average of the price and quantity relatives using arithmetic averages of the value shares in the two periods as weights, in other terms, they have the advantage to capture changes in the composition of the purchased basket. The TTD indices for prices and quantity are given by (1) and (2):

(1) $$\begin{array}{l} \frac{{\text{P}}_{\text{t}}}{{\text{P}}_{\text{t}-\text{1}}}\text{=}\prod_{\text{i=1}}^{\text{n}}{\left(\frac{{\text{p}}_{\text{i},\text{t}}}{{\text{p}}_{\text{i},\text{t}-\text{1}}}\right)}^{\frac{\text{1}}{\text{2}}\left[\frac{{\text{p}}_{\text{i},\text{t}-\text{1}}{\text{q}}_{\text{i},\text{t}-\text{1}}}{\sum_{\text{j=1}}^{\text{n}}\left({\text{p}}_{\text{j},\text{t}-\text{1}}{\text{q}}_{\text{j},\text{t}-\text{1}}\right)}\text{+}\frac{{\text{p}}_{\text{i},\text{t}}{\text{q}}_{\text{i},\text{t}}}{\sum_{\text{j=1}}^{\text{n}}\left({\text{p}}_{\text{j},\text{t}}{\text{q}}_{\text{j},\text{t}}\right)}\right]} \end{array}$$

(2) $$\begin{array}{l} \frac{{\text{Q}}_{\text{t}}}{{\text{Q}}_{\text{t}-\text{1}}}\text{=}\prod_{\text{i=1}}^{\text{n}}{\left(\frac{{\text{q}}_{\text{i},\text{t}}}{{\text{q}}_{\text{i},\text{t}-\text{1}}}\right)}^{\frac{\text{1}}{\text{2}}\left[\frac{{\text{p}}_{\text{i},\text{t}-\text{1}}{\text{q}}_{\text{i},\text{t}-\text{1}}}{\sum_{\text{j=1}}^{\text{n}}\left({\text{p}}_{\text{j},\text{t}-\text{1}}{\text{q}}_{\text{j},\text{t}-\text{1}}\right)}\text{+}\frac{{\text{p}}_{\text{i},\text{t}}{\text{q}}_{\text{i},\text{t}}}{\sum_{\text{j=1}}^{\text{n}}\left({\text{p}}_{\text{j},\text{t}}{\text{q}}_{\text{j},\text{t}}\right)}\right]} \end{array}$$

Where $\frac{P_{t}}{P_{t-1}}$ in (1) is the price index representing the average change in prices for the group in question (e.g., meat) from period t-1 to t; $\frac{p_{i,t}}{p_{i,t-1}}$ is the price index of product i (e.g., Aberdeen Angus beef steak price index, within the beef price calculation). *q*_*i,t*−1_ is the quantity of product i in period t-1; $\frac{p_{i,t-1}q_{i,t-1}}{\overset{n}{\underset{j=1}{\sum }}\left(p_{j,t-1}q_{j,t-1}\right)}$ and $\frac{p_{i,t}q_{i,t}}{\overset{n}{\underset{j=1}{\sum }}\left(p_{j,t}q_{j,t}\right)}$ are the expenditure shares of the product i (e.g., Aberdeen Angus beef steak) in the total expenditure of category (e.g., beef) in period t-1 and t. The formula in (2) is similar to (1) but in terms of quantities, where $\frac{Q_{t}}{Q_{t-1}}$ is the quantity index.

#### Purchases Decomposition

The construction of the indices was followed by analyzing the income groups response to changes in prices (*P*_*t*_), by changes in the expenditure (*E*_*it*_), in the quantities purchased (*Q*_*it*_) and in the quality purchased ($\frac{v_{it}}{P_{t}}$ i.e., trading up and down in quality), where *v*_*it*_ is a unit value ($\frac{E_{it}}{Q_{it}}$) (19, 20). For income group i in time period t, identity (3) was used to analyse the meat and fish purchase data:

(3) $${\text{E}}_{\text{it}}={\text{P}}_{\text{t}}\times {\text{Q}}_{\text{it}}\times \left(\frac{{\text{v}}_{\text{it}}}{{\text{P}}_{\text{t}}}\right)$$

Writing (3) in terms of indices that show the changes from period of t-1 to t one obtains (4):

(4) $$\frac{{\text{E}}_{\text{it}}}{{\text{E}}_{\text{it}-\text{1}}}\text{=}\frac{{\bar{\text{P}}}_{\text{t}}}{{\bar{\text{P}}}_{\text{t}-\text{1}}}\times \frac{{\text{Q}}_{\text{it}}}{{\text{Q}}_{\text{it}-\text{1}}}\times \frac{\left(\frac{{\text{v}}_{\text{it}}}{{\bar{\text{P}}}_{\text{t}}}\right)}{\left(\frac{{\text{v}}_{\text{it}-\text{1}}}{{\bar{\text{P}}}_{\text{t}-\text{1}}}\right)}$$

Expressing (4) in terms of rates of change we get (4')

(4') $$\left(1+\hat{E_{it}}\right)=\left(1+{\hat{\bar{P}}}_{t}\right)\times \left(1+\hat{Q_{it}}\right)\times \left(1+\hat{\frac{v_{it}}{{\bar{P}}_{t}}}\right)$$

Where the ‘^∧^’ indicates the rate of change of the variable with respect to a previous period (e.g., $\frac{x_{t}}{x_{t-1}}-1$). As explained by McKelvey (20), $\frac{v_{it}}{{\bar{P}}_{t}}$ is a measure of the quality of the group purchase. A higher expression means that the chosen *Q*_*it*_ is more expensive per unit of group consumption and this is interpreted as buying a higher quality (21).

The expression $\frac{v_{it}}{{\bar{P}}_{t}}$ can be further broken down as in expression (5), which multiplies the nutrient per quantity by the price of the nutrient in real terms. For this paper, the nutrient per quantity purchased is of particular interest because it is a measure of the nutritional quality of the purchased products. Note that this differs from the approach followed by Fousekis and Revell (13) where their notion of quality does not represent nutrition just meat substitutions.

(5) $$\frac{{\text{v}}_{\text{it}}}{{\bar{\text{P}}}_{\text{t}}}\text{=}\left(\frac{{\text{N}}_{\text{it}}}{{\text{Q}}_{\text{it}}}\right)\times \left(\frac{\frac{{\text{E}}_{\text{it}}}{{\bar{\text{P}}}_{\text{t}}}}{{\text{N}}_{\text{it}}}\right)$$

Where:

*E*_*it*_ = All meats expenditure in period t by group i

${\bar{P}}_{t}$ = Average price of meat in period t (i.e., over all groups)

*Q*_*it*_ = Total purchased quantity of meat in period t by group i

*N*_*it*_ = Total nutrient (e.g., calories) in period t by group i

#### Trend Analysis

The nutrient per quantity purchased was subject to a trend analysis. For this, the both the calories, saturated fats and sodium per quantity, were first seasonally adjusted before estimating the trends. This was done to avoid confuse any change during the COVID-19 period with a seasonal component. Based on the observed data graphs model (6) was estimated:

(6) $$\begin{array}{l} \frac{{\text{N}}_{\text{it}}}{{\text{Q}}_{\text{it}}}\;=\alpha_{\text{0}}{\text{+ β}}_{\text{0}}\times \text{t + }{\text{α}}_{\text{1}}\times {\text{d}}_{\text{1}}{\text{+ β}}_{\text{1}}\times {\text{d}}_{\text{1}}\times \text{ t }{\text{+ α}}_{\text{2}}\times {\text{d}}_{\text{2}}{\text{+ β}}_{\text{2}}\\ \;\times \;{\text{d}}_{2}\times \text{t} \end{array}$$

Where:

α_*i*_ = Intercept of the trend regression for the full period (i = 0,1,2)

β_*i*_ = Slope of the trend regression for the full period (i = 0,1,2)

*t* = Trend (defined as 0, 1, 2….)

*d*_1_ = binary variable that takes the value of 1 for the period 2018-01 to 2020-02

*d*_2_ = binary variable that takes the value of 1 for the period 2020-03 to 2020-07

Note that if α_**2**_ is statistically different than zero (using a *t*-test and 95 percent significance level), it means that the COVID-19 intercept of the trend regression line is equal to (α_**0**_+α_**2**_). Similarly, the slope of the regression line if β_**2**_ is statistically different than zero is equal to (β_**0**_+β_**2**_).

## Results

### Evolution of Aggregated Meat Expenditure, Prices and Purchased Quantities

**Figure 3:**
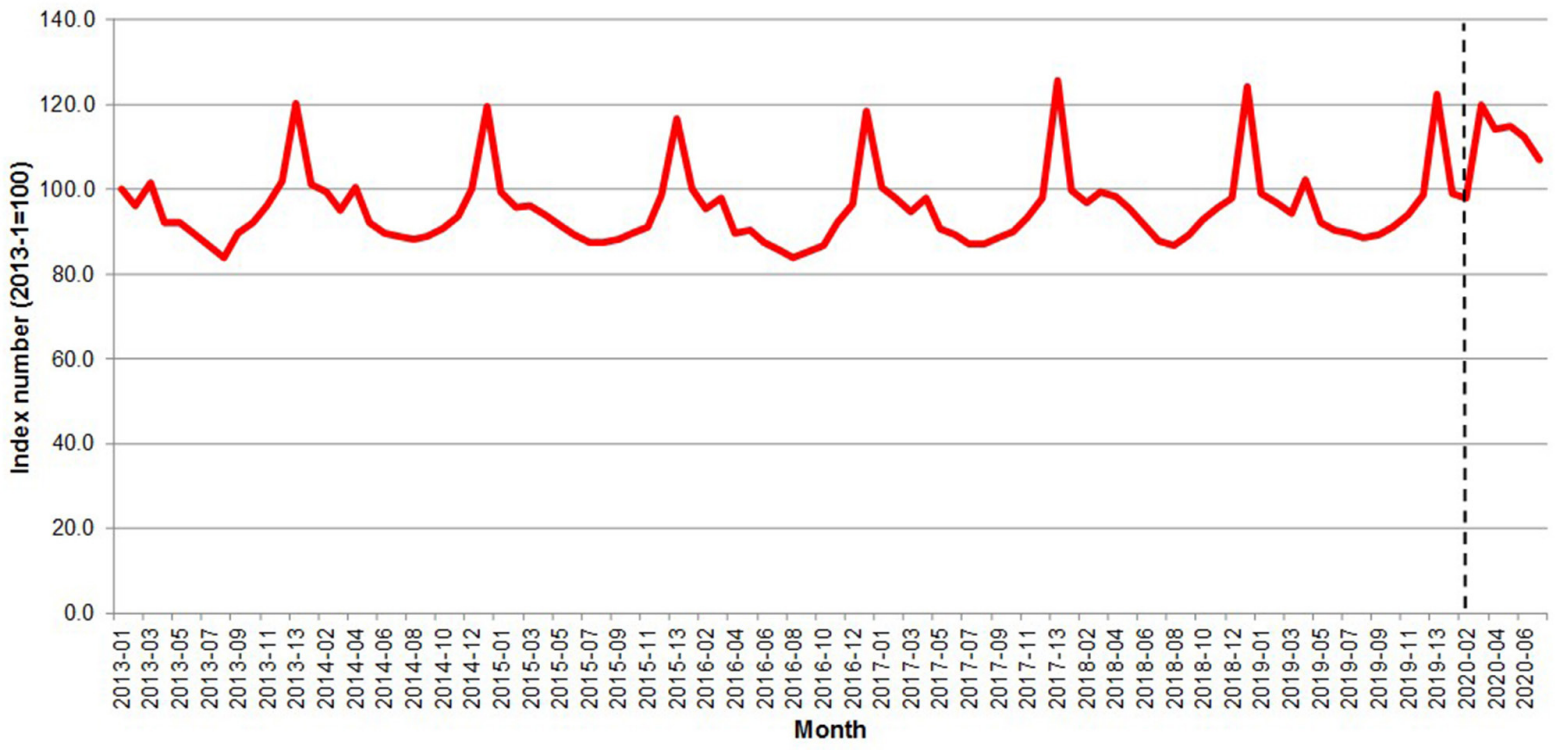
Great Britain—Evolution of the per capita meat expenditure 2013–2020. Source: Own elaboration based on Kantar Worldpanel data. Note: The dotted line represents the start of the lockdown in March 2020.

The pandemic emergency was associated with a sharp increase in expenditure for meat and fish products. Figure 3 shows that after the pandemic outbreak (early March 2020), the Great Britain expenditure for at-home purchase of meat and fish, instead of declining to the usual low-season level, jumped to a level that was comparable to the previous Christmas peak and slowly decreased thereafter.

During the outbreak period, when the purchasing surge was at its peak, a moderate price decrease was detected (0.74 percent). Prices of meat and fish were two percent higher than before the outbreak were observed starting from the t+1 period. This result is roughly consistent with the EU food inflation estimate by Akter (22) but much lower than the data by the US Bureau of Labor Statistics (23) reporting a 10.3 percent increase in the consumer price index in the meats, poultry, fish, and eggs category between March and June 2020.

**Figure 4:**
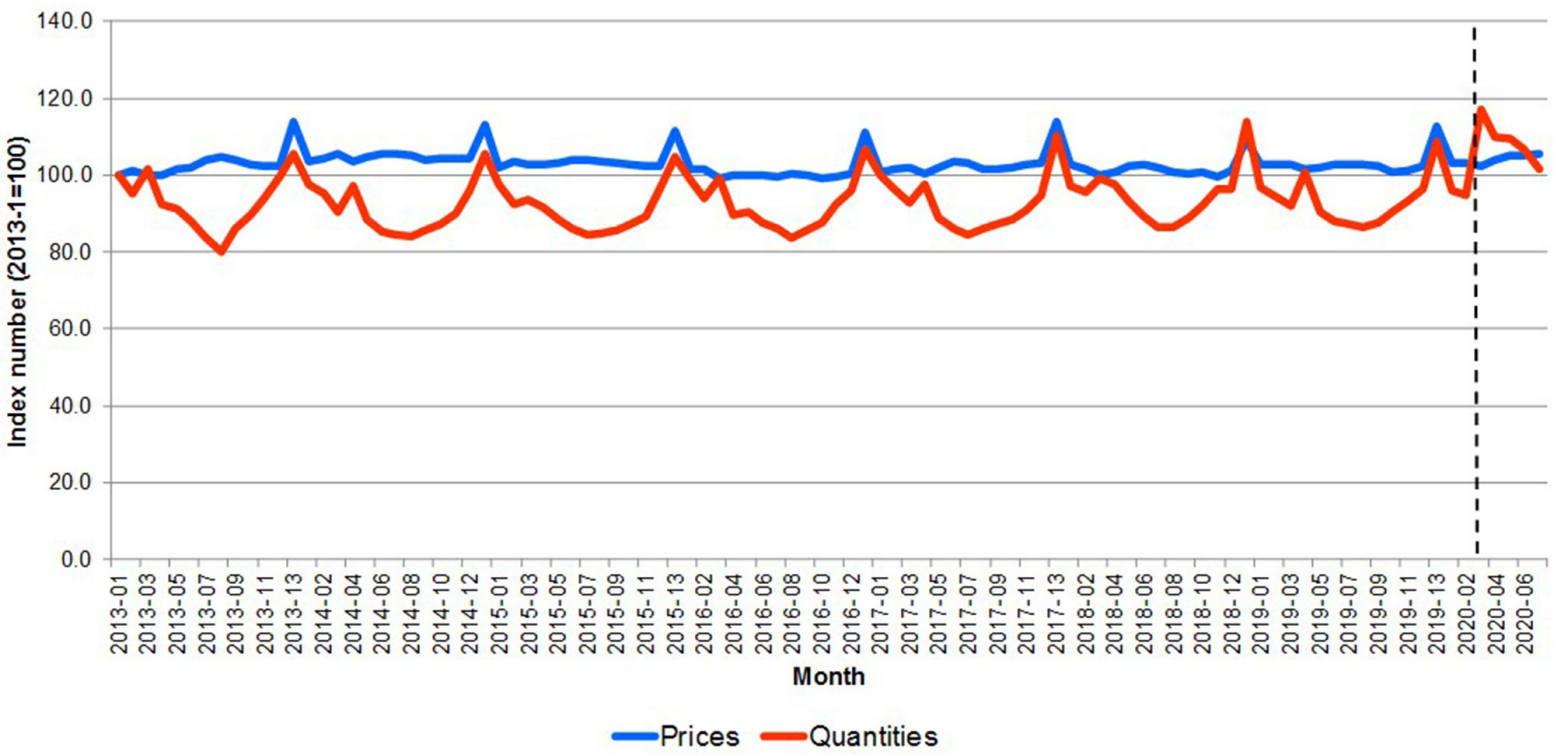
Great Britain—Evolution of the price and per capita meat quantity 2013–2020. Source: Own elaboration based on Kantar Worldpanel data. Note: The dotted line represents the start of the lockdown in March 2020.

Figure 4 shows the meat and fish aggregated price and quantity indices; the pandemic outbreak was associated with a rapid surge in quantity at the outbreak (22.4-point index increase) quickly reverting to the long-term average and with a much slower but persistent increase in prices (2.9-point index increase over the entire pandemic period). The result is consistent with the observed price rigidity in the pre-pandemic periods, when the variability in prices over time was much smaller than the one in quantities.

### Decomposition of Meat and Fish Expenditure

**Figure 5:**
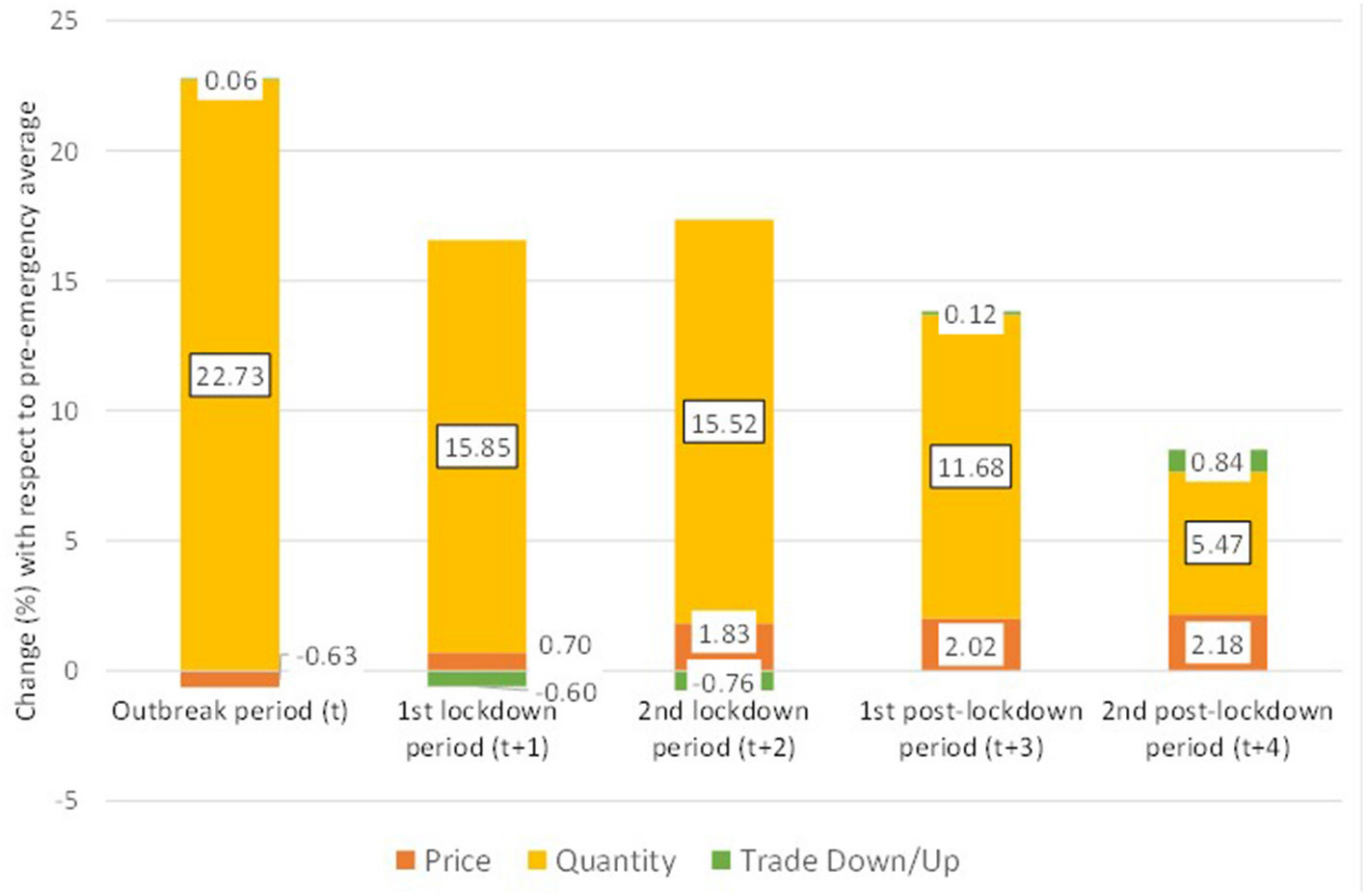
Great Britain—Decomposition of meat and fish expenditure after the pandemic outbreak. Note: Computed using the Deaton-McKelvey decomposition of meat and fish expenditure increase after the pandemic outbreak in the UK. All changes are computed with respect to the pre-emergency average meat and fish expenditure in Great Britain.

Using the Deaton (19) and McKelvey's (20) method the change in meat and fish expenditure with respect to the pre-emergency 2013-2020 average was decomposed into three components: price, quantity and quality (trading up or down). Figure 5 presents the results.

Figure 5 shows a major surge in meat and fish expenditure (22 percent) during the outbreak period, even before restrictive measures and lockdown were imposed. A possible explanation for this behavior is a stockpiling effect due to consumers' concerns for future shortage as well as shift toward home consumption due to the closure of the food service (7, 10). In this period, the overall increase was determined by the increase in purchase quantity (22.6 percent), while average prices decreased slightly (0.6 percent). The trade up/down effect was almost negligible (0.05 percent).

During the lockdown periods, the meat and fish expenditure was still higher than the pre-pandemic levels, but lower than during the outbreak period. The changes were +16 percent in period t+1 and +15.9 percent in period t+2. Quantity was the main driver of the change still (+15.9 in t+1 and +15.7 in t+2), yet a moderate average price increase was detected (+0.7 and +1.8 percent, respectively). In these periods, a small trade down effect was observed (−0.6 and −0.7 percent, respectively).

A possible explanation of the increase with respect to pre-pandemic level is consumer replacement of away-from-home food for food to consume at home while the decrease with respect to the outbreak period might be explained with the lower concerns about future shortage as consumers became aware of the stable food supply despite the pandemic.

In the post-lockdown periods, decreases in the expenditure for meat and fish were observed. The level was still higher than the pre-emergency average (+14.1 percent in t+3 and +8.7 in t+4), but lower than during the lockdown periods. The main driver was still the increased consumption, yet the average price levels were higher than in the pre-emergency period (+2.1 percent and +2.2 percent, respectively), and a moderate trade up effect was detected in period t+4 (+0.9 percent). The data from first 2 months after lockdown suggested a consumption trend slowly reverting to pre-pandemic behavior, and a persistence in a price increase of ~2 percent.

The changes in meat and fish expenditure were the result of heterogeneous trends in the consumption of different types of product. Table 1 reports the decomposition of expenditure for six product categories: beef, lamb, pork, poultry, other meat and fish. Poultry was the fastest category to revert to pre-emergency consumption level and registered the smallest variation in prices. On the other hand, fish exhibited a remarkable price surge starting in period t+1, a relatively small increase in consumption and a distinctive persistence of the change in consumer behavior. Pork and other meat categories exhibited fluctuating trends.

**Table 1:** Great Britain—Decomposition of the change in expenditure by meat and fish (%).

**Product**		**Outbreak period**								
		**ΔP**	**ΔE**	**ΔQ**	**T**					
Beef		−2.27	24.77	27.67	0.00					
Lamb		0.03	20.39	20.29	0.04					
Pork		−0.87	27.21	28.28	0.04					
Poultry		0.31	19.14	18.74	0.03					
Fish		−1.11	16.28	17.56	0.02					
Other meats		0.35	27.29	26.63	0.18					
**Product**		**Lockdown period 1**					**Lockdown period 2**			
		**ΔP**	**ΔE**	**ΔQ**	**T**		**ΔP**	**ΔE**	**ΔQ**	**T**
Beef		−3.69	17.95	22.36	0.09		0.84	20.32	19.21	0.09
Lamb		−2.39	64.82	68.58	0.16		1.87	8.99	6.83	0.14
Pork		2.97	20.68	17.13	0.07		3.06	27.87	24.08	0.00
Poultry		0.69	17.71	16.64	0.24		−0.79	12.58	13.21	0.24
Fish		3.15	−1.38	−4.70	0.32		4.95	9.90	4.49	0.21
Other meats		2.36	18.12	14.97	0.38		1.50	23.25	20.97	0.39
**Product**		**Post-lockdown period 1**					**Post-lockdown period 2**			
		**ΔP**	**ΔE**	**ΔQ**	**T**		**ΔP**	**ΔE**	**ΔQ**	**T**
Beef		0.06	12.37	12.15	0.13		2.89	9.63	6.44	0.10
Lamb		2.76	11.58	8.43	0.13		3.40	16.58	12.71	0.03
Pork		5.50	27.33	20.60	0.08		2.19	17.71	15.15	0.04
Poultry		−0.16	7.33	7.24	0.25		−0.35	−0.79	−0.65	0.22
Fish		5.61	14.96	8.62	0.21		5.34	11.16	5.39	0.12
Other meats		0.85	18.79	17.34	0.38		0.33	10.71	10.01	0.30

*Source: Own elaboration based on Deaton and McKelvey*.

*ΔE stands for the change in expenditure, ΔP is the change in price, ΔQ is the change in quantity and T is trading up/down effect by product after the pandemic outbreak in the UK. All changes are computed with respect to the pre-emergency averages in the UK*.

The analysis of quality trade-up/down found a moderate trade down when the entire (aggregated) category of meat and fish was considered (Figure 5). Instead, when specific types of products were considered, no trade-down effect was detected and a limited (0.38 at most) trade-up was found (Table 1). The results might be explained by cross-product substitution. Consumers willing to reduce expenditure bought cheaper types of meat (e.g., moving from fish to poultry) instead of buying cheaper cuts of the same meat.

**Figure 6:**
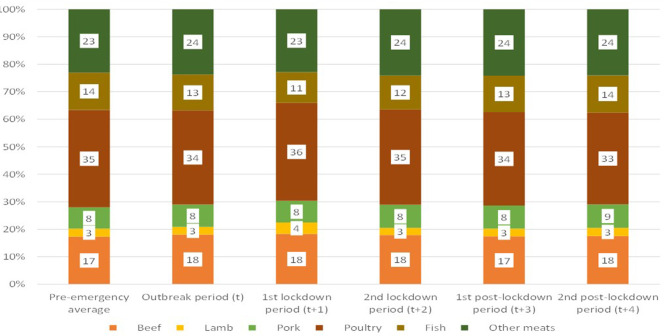
Great Britain—Composition of the meat and fish purchased quantities. Source: Own elaboration based on Kantar Worldpanel data.

Figure 6 reports the quantity shares of each type of meat in the aggregated consumer basket. The Figure shows very small changes during the lockdown periods with the shares remaining mostly constant, with changes at most of two percent points compared to the pre-pandemic composition.

**Figure 7:**
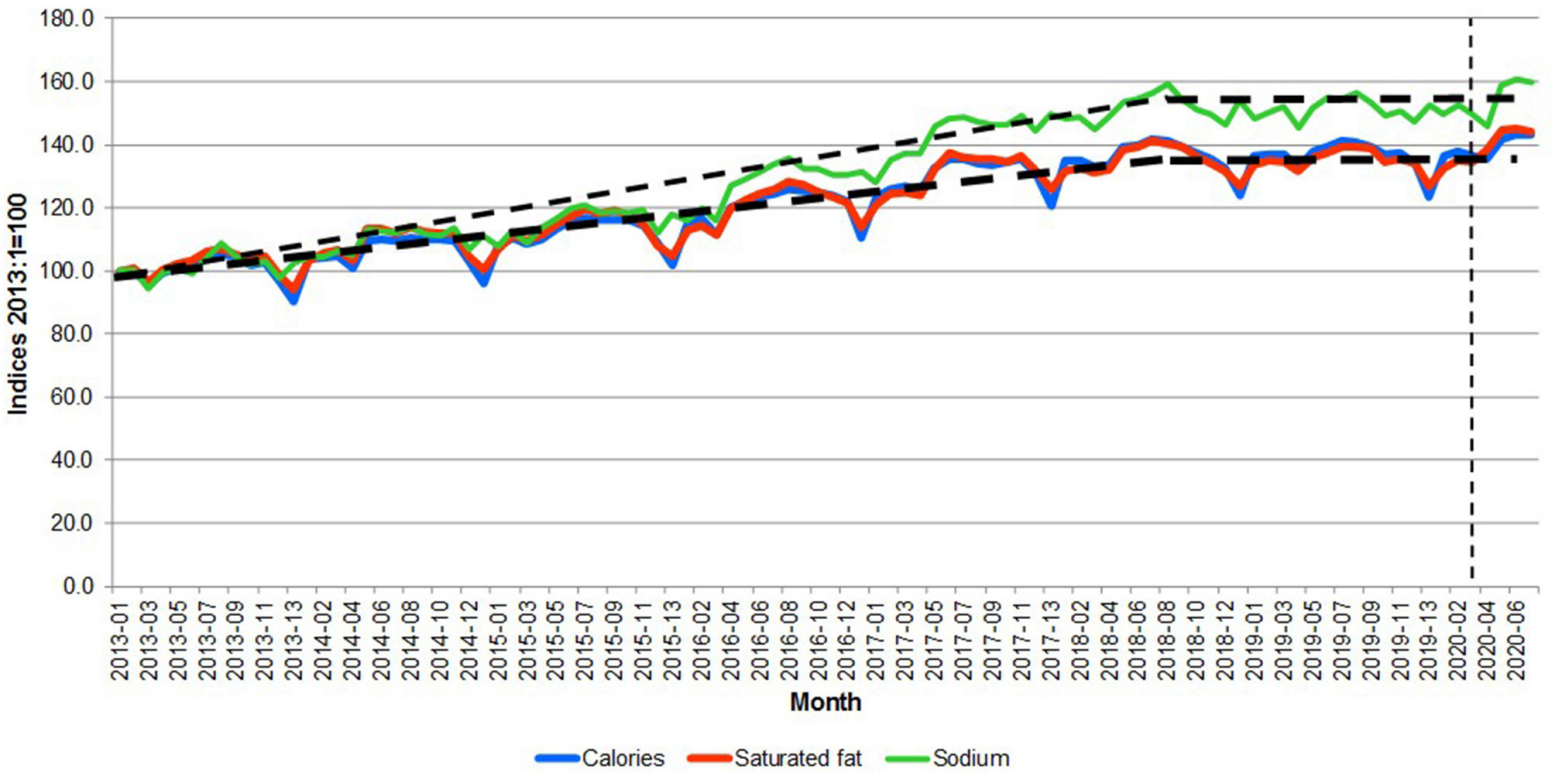
Great Britain—Calories, saturated fat and sodium content per kilogram of purchased meat and fish. Source: Own elaboration based on Kantar Worldpanel data. Note: The thin dotted line represents the start of the lockdown in March 2020. The thick dotted lines represent trends.

Figure 6 does not show the impact of the composition of the meat and fish basket on nutrition. In order to illustrate this effect, Figure 7 reports calories and saturated fat in the consumer meat and fish basket.

Figure 7 shows three potential periods, namely: 2013-01 to 2017-13, 2018-01 to 2020-02 and 2020-03 onwards. The data indicate during lockdown, the substitution of fish and poultry toward other meat products determined a shift toward less healthy meat consumption (+2.0 percent, +5.5 percent +2.5 percent in a month for saturated fats, energy and sodium, respectively). However, the effect is attenuated in the post-lockdown periods, suggesting a transitory effect and a possible reversion to the long-run trend after the pandemic.

### Analysis by Income Group

In this section the effects of the pandemic shocks are investigated by computing the decomposition of the change in meat and fish expenditure by household income group. Table 2 reports the results of the analysis, showing remarkable differences in behavior.

**Table 2:** Great Britain—Decomposition of the change in expenditure for meat and fish by income group (%).

**Income group**		**Outbreak period (t)**								
		**ΔP**	**ΔE**	**ΔQ**	**T**					
<£ 30,000		−0.63	19.99	19.74	0.85					
£30,000–39,999		−0.63	24.06	25.74	−0.70					
£40,000–49,999		−0.63	24.01	24.74	0.02					
£50,000–59,999		−0.63	22.29	22.03	0.86					
Over £60,000		−0.63	20.62	23.08	−1.37					
**Income group**		**Lockdown period 1 (t+1)**					**Lockdown period 2 (t+2)**			
		**ΔP**	**ΔE**	**ΔQ**	**T**		**ΔP**	**ΔE**	**ΔQ**	**T**
<£ 30,000		0.70	8.48	7.46	0.24		1.83	9.37	6.71	0.65
£30,000–39,999		0.70	11.18	12.26	−1.65		1.83	14.64	14.43	−1.61
£40,000–49,999		0.70	23.00	24.00	−1.53		1.83	23.26	22.46	−1.18
£50,000–59,999		0.70	26.07	25.29	−0.08		1.83	26.07	24.93	−0.90
Over £60,000		0.70	29.38	29.36	−0.68		1.83	26.04	24.87	−0.87
**Income group**		**Post-lockdown period 1 (t+3)**					**Post-lockdown period 2 (t+4)**			
		**ΔP**	**ΔE**	**ΔQ**	**T**		**ΔP**	**ΔE**	**ΔQ**	**T**
<£ 30,000		2.02	6.75	3.34	1.25		2.18	1.74	−2.11	1.71
£30,000–39,999		2.02	12.90	10.73	−0.06		2.18	6.43	3.03	1.09
£40,000–49,999		2.02	20.41	18.61	−0.52		2.18	12.27	8.14	1.57
£50,000–59,999		2.02	19.45	18.32	−1.05		2.18	15.18	11.96	0.68
Over £60,000		2.02	25.81	23.45	−0.10		2.18	17.78	13.77	1.32

*Source: Own elaboration based on Deaton and McKelvey*.

*ΔE stands for the change in expenditure, ΔP is the change in price, ΔQ is the change in quantity and T is trading up/down effect by product after the pandemic outbreak in the UK. All changes are computed with respect to the pre-emergency averages in the UK*.

Although all group exhibited similar reactions during the outbreak period, the behavior during and after the lockdown diverged. Starting from period t+1, consumers in the lowest income group (<£30,000 per year) exhibited a moderate increase in consumption and a trading-up effect. Instead, households in the highest income groups (over £ 50,000) increased their consumption by 25 percent in period t+1, slowly declining to a +14 percent in period t+4. They exhibited a limited trading-down effect, which was compensated in part by trading-up in period t+4. Middle class groups (between £30,000 and 49,999) were characterized by the deepest trading-down effect.

The decomposition showed that the change in expenditure for meat and fish was explained mainly by variations in quantity, while price and trading-up or down effects were limited. The main differences across income groups concerned the magnitude of the quantity adjustments and the direction of the trading-up/down effects. As expected, lower income groups exhibited higher price sensitivity. Households in the <£-30,000 income group increased consumption largely during the outbreak period, when prices were lower than the pre-emergency average and the stockpiling effect was at its peak. In the following periods, as prices increased, consumption decreased. Finally, in period t+4, the group consumption fell below the pre-emergency average quantity. The consumption of high-income groups (£ 50,000 and above) did not exhibit such direct association with prices. Instead, it was driven by the movement restrictions. It increased during the outbreak period, peaked during the lockdown and slowly declined afterward as movement restrictions were eased and the fear of contagion. Noticeably, the impact on quantity for high-income groups seems to be associated with the psychological pressure of the contagion, while the additional effect of the restrictions was limited (10).

**Figure 8:**
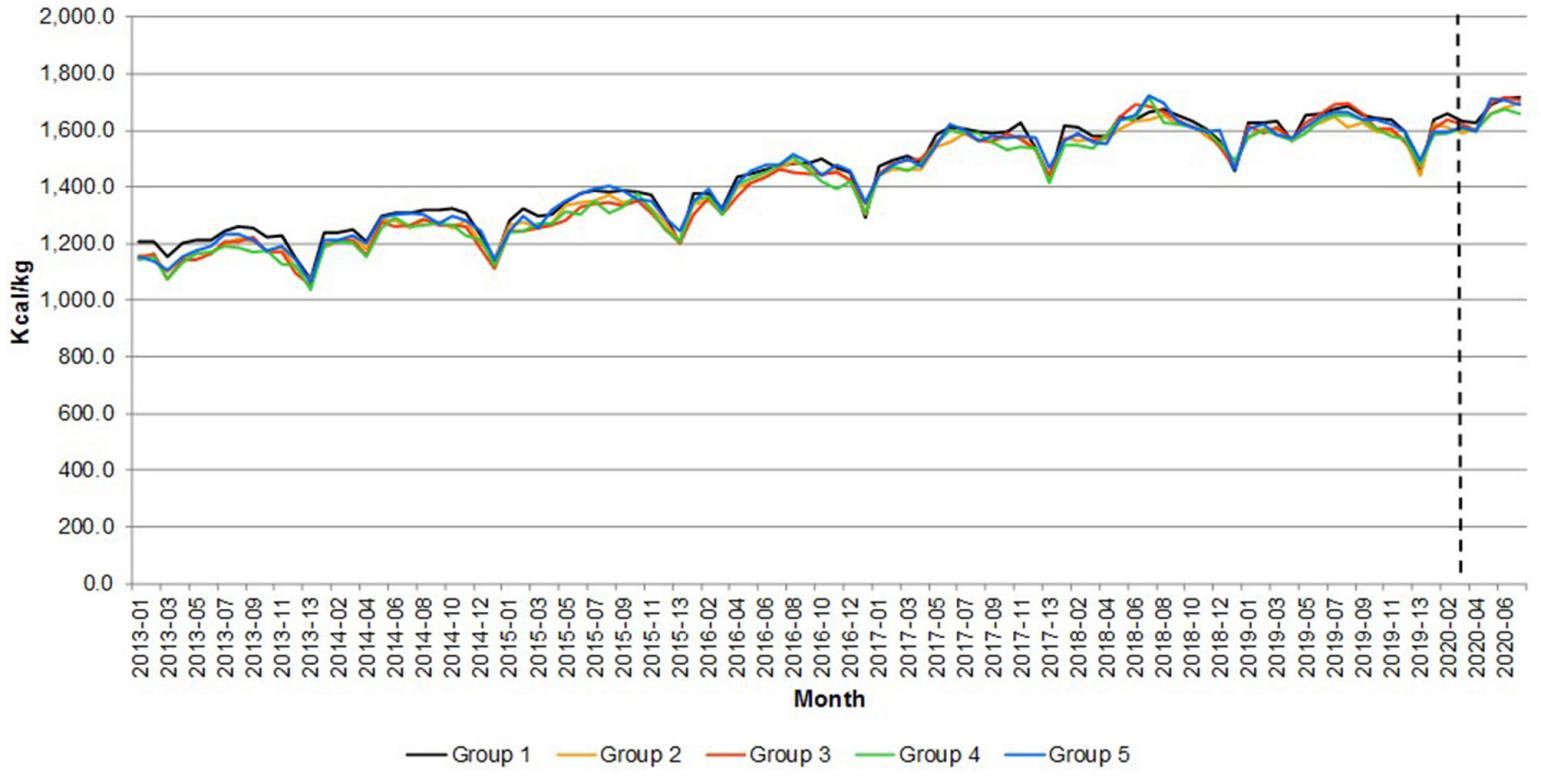
Great Britain—Calories per quantity of meat and fish. Source: Own elaboration based on Kantar Worldpanel data. Note: The dotted line represents the start of the lockdown in March 2020. Group 1 = <£30,000, Group 2 = £30,000–39,999, Group 3 = £40,000–49,999, Group 4 = £50,000–59,999, and Group 5 = over £60,000.

**Figure 9:**
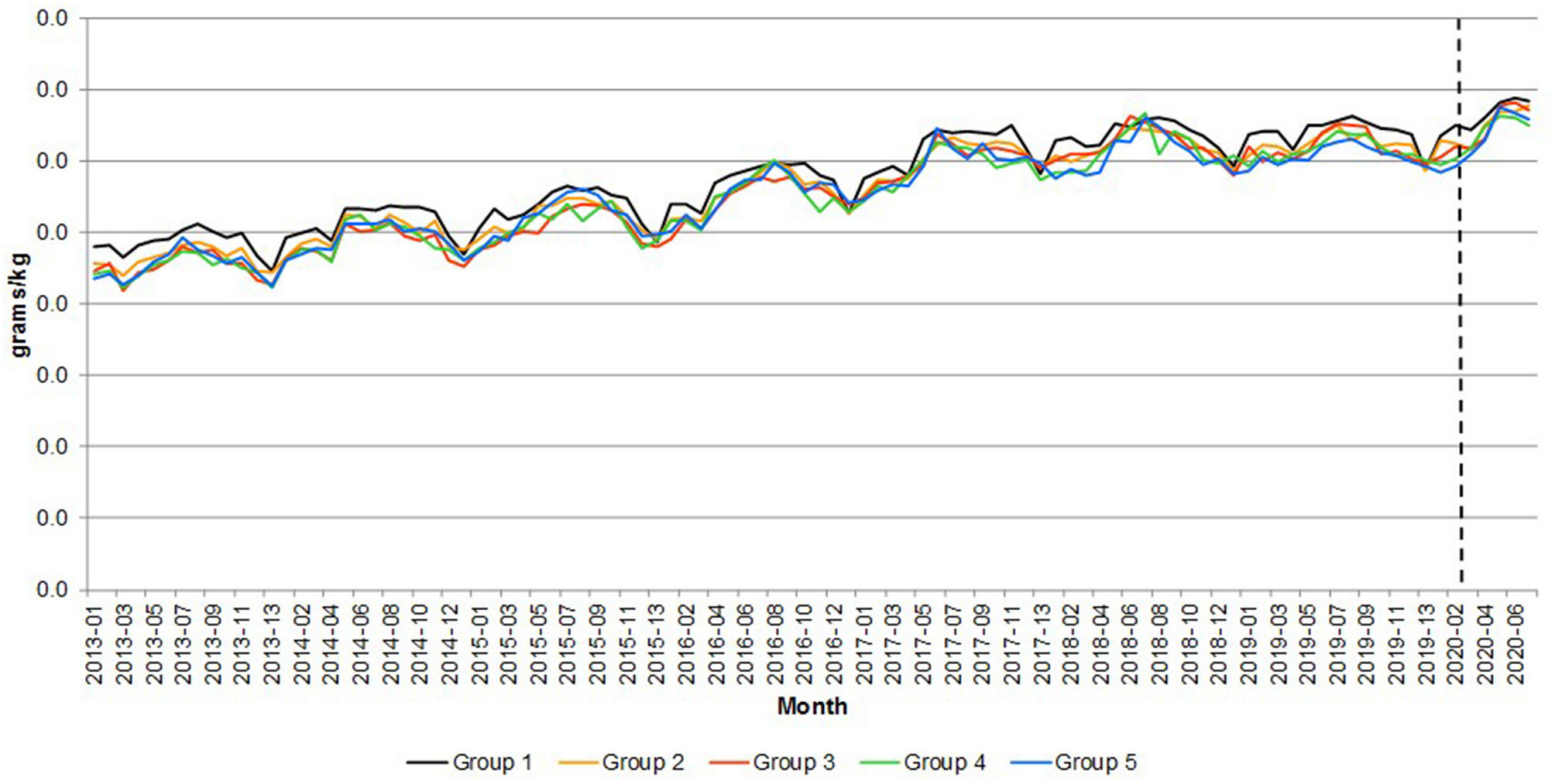
Great Britain—Saturated fats per quantity of meat and fish. Source: Own elaboration based on Kantar Worldpanel data. Note: The dotted line represents the start of the lockdown in March 2020. Group 1 = <£30,000, Group 2 = £30,000–39,999, Group 3 = £40,000–49,999, Group 4 = £50,000–59,999, and Group 5 = over £60,000.

**Figure 10:**
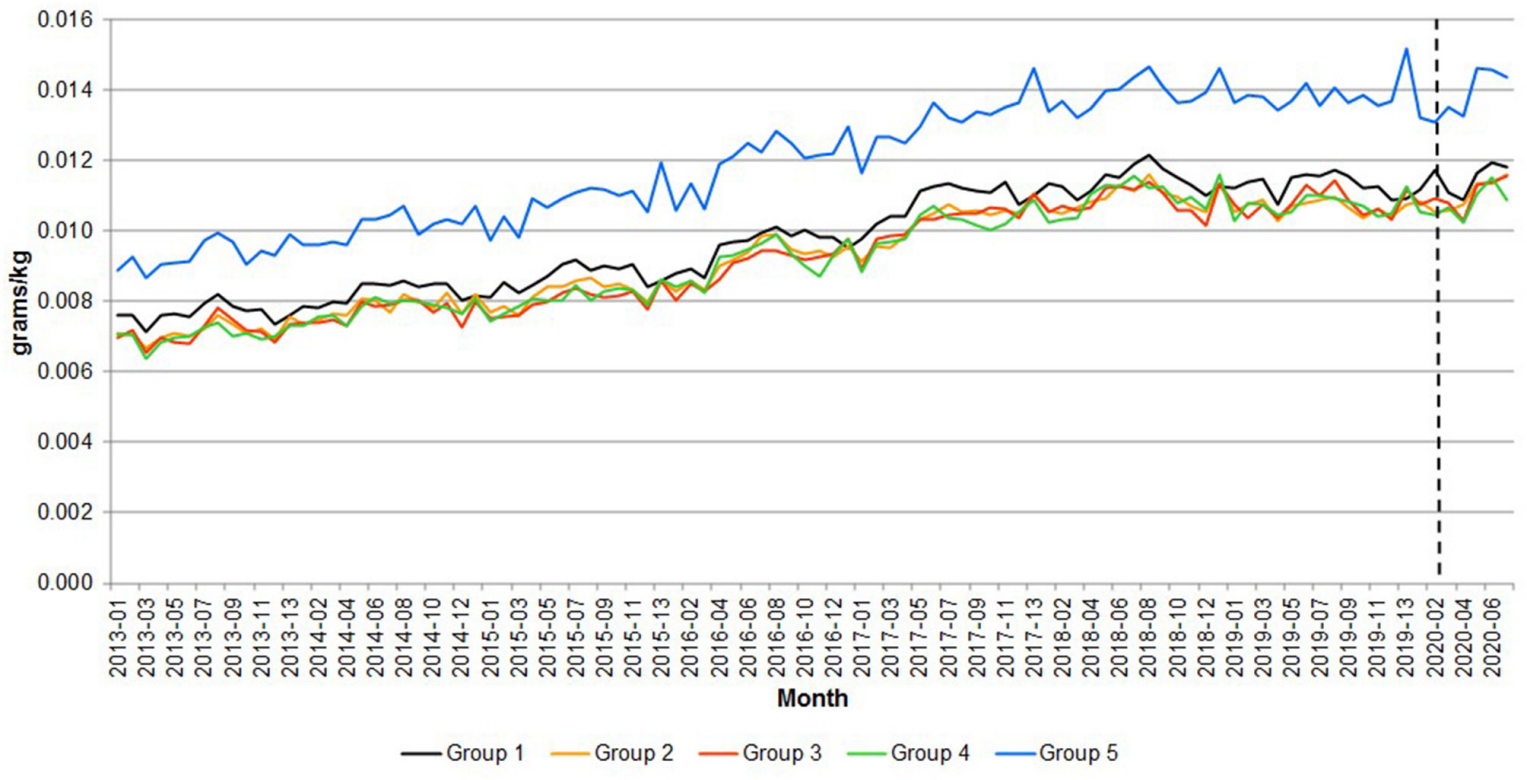
Great Britain—Sodium per quantity of meat and fish. Source: Own elaboration based on Kantar Worldpanel data. Note: The dotted line represents the start of the lockdown in March 2020. Group 1 = <£30,000, Group 2 = £30,000–39,999, Group 3 = £40,000–49,999, Group 4 = £50,000–59,999, and Group 5 = over £60,000.

The change in consumption behavior had an impact on nutrition. Figures 8–10 compare the calories, saturated fat and sodium per kilogram of meat and fish by income group, respectively. The pandemic emergency was associated with an increase in calories, saturated fat showing that all groups moved to more caloric and less healthy meat and fish purchases. On average, both calories and saturated fat per kilogram have been growing at a monthly rate of 0.37 percent from 2013 to 2020. Figure 10 shows that the amount of sodium per kilogram also increased over time (it grew 0.48 percent per month from 2013 to 2020). Particularly interesting is that the most affluent group (over £60,000) purchased meat and fish with more sodium per kilogram (~30 percent more than the average of the other income groups).

Table 3 reports the percentage change in the average calories, saturated fat per kilogram and sodium of meat and fish in the emergency periods with respect to the same periods in 2019, in order to account for seasonality in consumption.

**Table 3:** Great Britain—Calories, saturated fats and sodium per kilogram of meat and fish (12-month percent change).

**Income group**	**Per-kg average calories**				
	**Outbreak**	**Lockdown 1**	**Lockdown 2**	**Post-lockdown 1**	**Post-lockdown 2**
<£ 30,000	−0.14	4.07	2.52	3.43	2.30
£30,000–39,999	−0.87	2.74	3.14	3.19	2.89
£40,000–49,999	0.59	1.84	4.24	3.60	0.94
£50,000–59,999	1.30	2.61	4.10	2.45	0.43
Over £60,000	1.46	1.56	6.28	3.46	1.69
**Income group**	**Per-kg saturated fats**				
	**Outbreak**	**Lockdown 1**	**Lockdown 2**	**Post-lockdown 1**	**Post-lockdown 2**
<£ 30,000	0.06	7.32	4.94	5.80	4.28
£30,000–39,999	−0.57	6.50	7.21	4.85	4.19
£40,000–49,999	1.32	4.75	10.32	6.50	3.03
£50,000–59,999	3.25	6.27	8.02	5.69	1.29
Over £60,000	2.55	4.26	12.37	7.55	5.06
**Income group**	**Per-kg sodium**				
	**Outbreak**	**Lockdown 1**	**Lockdown 2**	**Post-lockdown 1**	**Post-lockdown 2**
<£ 30,000	−3.06	0.95	1.32	2.88	2.10
£30,000–39,999	−2.99	4.27	6.08	5.15	6.51
£40,000–49,999	0.29	−0.92	5.26	0.66	4.80
£50,000–59,999	−0.94	−2.09	4.65	4.31	−1.34
Over £60,000	−2.10	−1.20	6.65	2.92	5.81

*Source: Own elaboration based on Kantar Worldpanel data*.

The data highlight differences between the lowest income group and the other ones. The former exhibited a sharp surge in the variables during the first lockdown period and a moderate increase compared to previous year afterwards. Households with income equal or higher than £30,000 registered the peak increase during the second lockdown period and a slow decline afterwards in the rate of change. Noticeably, the higher income groups seem to be faster in reverting to the previous year nutrition behavior than lower income groups (with a partial exception for households with income over £60,000).

The results suggest that nutrition consequences of the lockdown were more severe for higher income groups than other ones, as far home meat and fish consumption was concerned. These households registered high increase in consumption of saturated fat and calories from those categories. This result is consistent with the large increase in meat purchases during the pandemic emergency that was observed for these groups.

### Trend Analysis of Calories and Saturated Fats per Quantity of Meat and Fish Purchased

Table 4 presents the results of the trend analysis of calories, saturated fats and sodium per quantity by income group. Recall that the purpose of this analysis is to explore whether the trend during the COVID-19 period (i.e., since 2020-03) was different to the trend considering the full period.

**Table 4:** Great Britain—Trend regression analysis of calories and saturated fats per purchased kilogram for all meats and fish.

	**Full sample**				**2018–2020-02**				**Covid**				***R*^**2**^**
	**Intercept**		**Trend**		**Intercept**		**Trend**		**Intercept**		**Trend**		
**<** **£ 30,000**													
Calories	−298.5		6.5		389.5		−5.5		437.7		−5.8		0.98
	−48.9*		39.5*		8.4*		−9.0*		0.6		−0.7		
Saturated fats 2/	−5.5		0.1		7.2		−0.1		9.7		−0.1		0.97
	−41.6*		33.7*		7.1*		−7.8*		0.6		−0.7		
Sodium 2/	−2.7		0.1		4.6		−0.1		0.5		0.0		0.96
	−38.4*		31.2*		8.6*		−9.1*		0.1		−0.2		
**£30,000–39,999**													
Calories	−309.1		6.8		442.5		−6.3		171.6		−3.2		0.98
	−53.5*		43.7*		10.1*		−10.9*		0.2		−0.4		
Saturated fats 2/	−5.7		0.1		7.4		−0.1		0.4		0.0		0.97
	−42.2*		34.8*		7.2*		−8.1*		0.0		−0.1		
Sodium 2/	−2.6		0.1		5.1		−0.1		−3.3		0.0		0.97
	−40.4*		33.0*		10.3*		−10.9*		−0.4		0.3		
**£40,000–49,999**													
Calories	−328.3		7.1		455.0		−6.3		587.3		−7.5		0.98
	−46.4*		37.4*		8.5*		−9.0*		0.7		−0.8		
Saturated fats 2/	−6.1		0.1		9.4		−0.1		2.2		0.0		0.96
	−38.7*		31.7*		7.8*		−8.5*		0.1		−0.2		
Sodium 2/	−2.7		0.1		4.4		−0.1		−3.0		0.0		0.96
	−34.9*		28.1*		7.6*		−7.9*		−0.3		0.2		
**£50,000–59,999**													
Calories	−321.7		7.1		467.6		−6.6		1551.3		−17.9		0.98
	−47.8*		39.1*		9.2*		−9.9*		1.9		−2.1*		
Saturated fats 2/	−5.9		0.1		7.6		−0.1		38.6		−0.4		0.96
	−36.8*		30.2*		6.3*		−7.1*		2.0		−2.1*		
Sodium 2/	−2.6		0.1		4.9		−0.1		6.3		−0.1		0.96
	−35.6*		28.6*		8.8*		−9.0*		0.7		−0.8		
**Over £60,000**													
Calories	−314.6		7.0		453.0		−6.5		935.0		−11.3		0.98
	−53.6*		44.2*		10.2*		−11.2*		1.3		−1.5		
Saturated fats 2/	−5.7		0.1		8.2		−0.1		17.3		−0.2		0.96
	−39.5*		33.5*		7.5*		−8.9*		1.0		−1.1		
Sodium 2/	−3.3		0.1		5.7		−0.1		−0.7		0.0		0.97
	−40.6*		33.4*		9.3*		−10.0*		−0.1		−0.1		

1/ First rows are the coefficients and the second rows are the standard error of the coefficients. *indicates that the coefficient is statistically different from zero at 95% significance.

2/ Coefficient multiplied by 1,000. Second row are t statistics. *indicates that the coefficient is statistically different from zero at 95% significance.

As shown in the Table, excepting in the case of the £50,000–59,999 group, which show a decreasing trend both in terms of calories and saturated fats (with statistically significant t statistics), all the other income groups show that the estimated trend using the full sample is appropriate for the COVID-19 period. Also note that this is not the case for the 2018-01 to 2020-02, which showed a line almost flat (i.e., no trend) as shown in Figure 7 for all the meats together.

**Figure 11:**
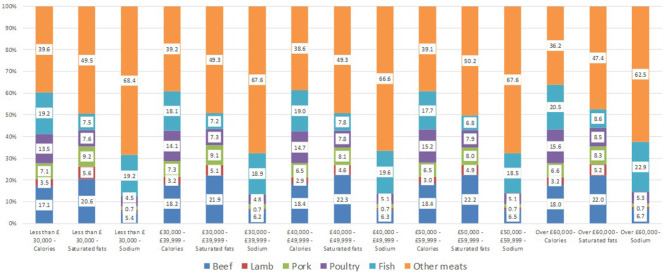
Great Britain—Average contribution of meat and fish to calories, saturated fats and sodium by income group during the COVID-19 period (2020-03 to 2020-07). Source: Own elaboration based on Kantar Worldpanel data.

The trend analysis of Table 4 is reflected in the composition of calories, saturated fats and sodium by meat and fish and income group presented in Figure 11. As shown in the figure, “other meats” has a significant contribution to calories, saturated fats and particularly to sodium for all the income groups. In the case of calories, the ‘other meats’ represent on average 39 percent of the total calories, 49 percent of the total saturated fats and 64 percent of the total sodium coming from meat and fish purchases.

## Discussion

The purpose of this study has been to study consumers reaction in terms of their purchases of meat and fish during the COVID-19 period using time series constructed from a scanner panel dataset for Great Britain by income groups.

It is important to note that the increase in the demand for particular meat cuts (e.g., mincemeat), the lack of demand from the food service and mild supply issues due to workers in processing plants contracting the Covid-19 virus created a temporary problem for the industry affecting the carcass balance, the demand for high quality meat and labor shortage, respectively. However, these now appear to have been solved as the industry has responded increasing the volumes delivered to supermarkets and recruiting additional labor force (24).

In aggregated terms, results indicated that consumers reacted initially to the lockdown by increasing their expenditure on at-home consumption of meat and fish, which was derived from a significant increase of the purchased quantities (10). This is consistent with previous demand analyses that indicated that meats had positive income elasticities [e.g., (13)]. Prices, in contrast, remained almost unchanged despite the demand pressure. Even more, the shares (computed from quantities) of the different meat and fish products remained very similar over time.

The results of the decomposition show the reaction of the UK meat and fish chain to an unexpected shock of unprecedented magnitude. The pandemic outbreak was associated to an immediate surge in meat and fish purchases. The increase might be explained by three effects: movement restriction, reduction of social interaction and stockpiling. Movement restrictions and spontaneous reduction of social interaction determined the substitution of food services (such as restaurants and catering) with at-home consumption. Also, uncertainty fear of shortage might trigger stockpiling behavior (10, 25). Nicola et al. (26) reported that “panic-buying has resulted in an increase of £1bn worth of food in UK homes.”

The combination of the three effects might explain the surge in home consumption during the outbreak period, and the declining trends thereafter. In the outbreak period stockpiling and voluntary reduction of social interaction took place, resulting in the largest increase in purchase. In the lockdown period the stockpiling effect faded, as home stocks were full and the perceived risk of food supply disruption declined, but movement restriction sustained home consumption as a substitute for away-from-home meals. In the post-lockdown periods, only the voluntary reduction of social interaction remained, and consumption trends slowly were reverting to pre-emergency levels.

It is important to note that the industry reacted quickly to the effects created by the lockdown (e.g., demand for cheaper meat cuts, lack of demand of the food service industry) by expanding the supply to supermarkets and also improving labor safety in the workplace (10). The price time series reflects this trend.

The evolution of the nutrients per quantity purchased (using calories and saturated fats as the chosen nutrients), which can be considered a measure of the nutritional quality of the purchases, indicated an increasing trend, which is consistent with the COVID-19 period. This contrasts with the period 2018 to 2020 before the lockdown, where there was almost no trend. Moreover, the quality of the meat and fish purchases is represented by the fact that the group “other meats” represented on average 39 percent of the calories contributed by meat and fish and 49 percent of the saturated fats during the period lockdown period.

The above results highlight the importance of emphasizing consumers' education and information as regards meat purchases. In this sense, NHS (1) recommendations pointing out at choosing lean cuts; if buying pre-packed meat to check the nutrition label to see the level of fat content, to purchase turkey and chicken without the skin as these are lower in fat (or remove the skin before cooking) and to limit processed meat products (other meats in the studied dataset) such as sausages, salami, pâté are very important.

## Data Availability Statement

The data used in the paper may be available upon request to the author.

## Author Contributions

CR-G: conceptualization, data preparation, investigation, visualization, formal analysis, writing—original draft, writing—review, and editing. CR: conceptualization, investigation, writing—review, and editing. Both authors contributed to the article and approved the submitted version.

## Conflict of Interest

The authors declare that the research was conducted in the absence of any commercial or financial relationships that could be construed as a potential conflict of interest.
